# Supplementary figures and images for: miR-3065-5p and miR-26a-5p as Clinical Biomarkers in Colorectal Cancer: A Translational Study
Source: Cancers (Basel). 2024 Oct 29;16(21):3649. doi: 10.3390/cancers16213649 (PMC11545460; doi:10.3390/cancers16213649)

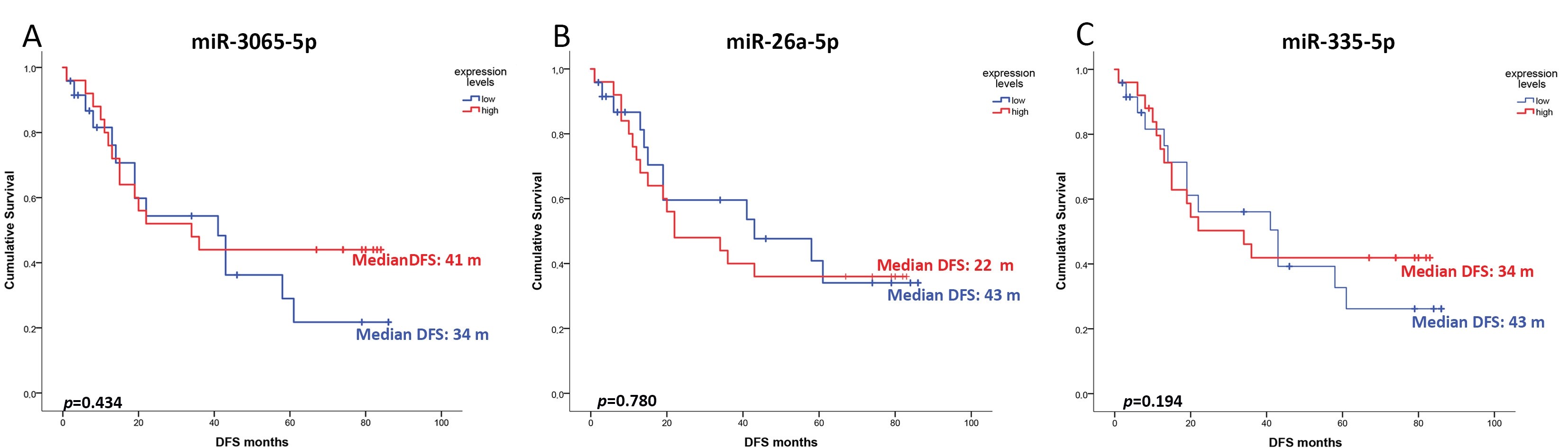

Supplement: Supplementary file 1 [file cancers-16-03649-s001.zip › cancers-3257255-supplementary.jpg]
